# Lipid Composition of Oil Extracted from Wasted Norway Lobster (*Nephrops norvegicus*) Heads and Comparison with Oil Extracted from Antarctic Krill (*Euphasia superba*)

**DOI:** 10.3390/md14120219

**Published:** 2016-12-01

**Authors:** Amaya Albalat, Lauren E. Nadler, Nicholas Foo, James R. Dick, Andrew J. R. Watts, Heather Philp, Douglas M. Neil, Oscar Monroig

**Affiliations:** 1 Institute of Aquaculture, Faculty of Natural Sciences, University of Stirling, Stirling FK9 4LA, UK; nicholasfoo93@gmail.com (N.F.); j.r.dick@stir.ac.uk (J.R.D.); oscar.monroig@stir.ac.uk (O.M.); 2 Institute of Biodiversity, Animal Health and Comparative Medicine, College of Medical, Veterinary and Life Sciences, Graham Kerr Building, University of Glasgow, Glasgow G12 8QQ, UK; lauren.nadler@my.jcu.edu.au (L.E.N.); a.watts.research@gmail.com (A.J.R.W.); Douglas.Neil@glasgow.ac.uk (D.M.N.); 3 Faculty of Life and Environmental Sciences, University of Iceland, Reykjavik 101, Iceland; heather_philp@yahoo.co.uk

**Keywords:** Norway lobster, *Nephrops norvegicus*, head waste, lipid class, EPA, DHA

## Abstract

In the UK, the Norway lobster (*Nephrops norvegicus*) supports its most important shellfish fishery. *Nephrops* are sold either whole, or as “tails-only” for the scampi trade. In the “tailing” process, the “head” (cephalothorax) is discarded as waste. A smaller crustacean species, the Antarctic krill *Euphasia superba*, represents an economically valuable industry, as its extractable oil is sold as a human dietary supplement. The aim of this study was to determine the amount and composition of the oil contained in discarded *Nephrops* heads and to compare its composition to the oil extracted from krill. Differences due to Geographical variation and seasonal patterns in the amount and composition of lipid were also noted. Results indicated that *Nephrops* head waste samples collected from more southern locations in Scotland (Clyde Sea area) contained higher levels of oil when compared to samples collected from northern locations in Iceland. Moreover, seasonal differences within the Clyde Sea area in Scotland were also observed, with oil extracted from *Nephrops* head waste peaking at around 11.5% during the summer months when larger and more mature females were caught by trawl. At this time of the year, the valuable fatty acids eicosapentaenoic acid (EPA) and docosahexaenoic acid (DHA) accounted for around 23% of the total fatty acid content in oil extracted from *Nephrops* head waste. A seasonal effect on EPA content was found, with higher levels obtained in the summer, while no trend was found in DHA percentages. Finally, oil from *Nephrops* head waste contained a higher proportion of EPA and DHA than krill oil but these fatty acids were more abundantly linked to the neutral lipids rather to than polar lipids. The characterization of lipid that could be extracted from *Nephrops* head waste should be seen as a first step for the commercial use of a valuable resource currently wasted. This approach is extremely relevant given the current limited supply of EPA and DHA and changes in the Common Fisheries Policy.

## 1. Introduction

The Norway lobster (*Nephrops norvegicus*), a decapod crustacean also known as the Dublin Bay prawn or langoustine, represents the second most valuable fishery in Scotland, being worth £61.7 million at first sale in 2013 [1]. Advances in the processing sector have established a market for a portion of this catch, whereby the tails (which contain the majority of edible meat) are detached from the “heads” (cephalothorax) and sold as “scampi” [2]. This “tailing” process is normally carried out on board the fishing vessels, with the “head” portions of these individuals disposed of at sea. Recently, the appropriate treatment of fisheries’ discards and waste products has become a pressing environmental issue with the passing of amendments to the Common Fisheries Policy (CFP) in the European Union, which is imposing a progressive ban on fisheries discards, with full implementation by 2019 [3]. Thus, the practice of disposing of *Nephrops* heads and claws at sea following tailing could be prohibited at some time in the near future. This change will therefore require innovative measures to be developed to dispose of or utilise the discards and waste products generated from *Nephrops* processing [3,4].

In contrast to other shellfish wastes, which comprise mainly chitin-rich shell material, the discarded heads from the *Nephrops* fishery contain the hepatopancreas and reproductive organs (gonads) in addition to the shell, both of which have been reported to contain very high levels of lipids [5]. Oils from other crustaceans, such as Antarctic krill (*Euphasia superba*), represent a good source of long-chain (>C_20_) polyunsaturated fatty acids (PUFAs), with significant amounts of docosahexaenoic acid (DHA) that are similar to those found in oily fish, but with generally higher concomitant contents of eicosapentaenoic acid (EPA) [6]. Furthermore, around 30%–60% of the fatty acids from krill oil comprise phospholipids (PL) in contrast to fish oil in which the fatty acids are stored mainly as triacylglycerols (TAG). This is of importance since krill oil has been shown to exhibit the same or enhanced positive health benefits as fish oils [7]. The reasons behind this are still unclear [8]. Studies suggest that the metabolic fate of key fatty acids like DHA differs when ingested as TAG, when compared to PL such as phophatidylcholine, especially in terms of bioavailability in plasma [9,10].

*Nephrops* has a wide geographical distribution, ranging as far north as Iceland and as far south as Mauritania, with fisheries throughout this range. The lipid amount and composition of the head waste may vary based on the geographical location of the fishery, due to variability in the lipid content of the hepatopancreas and gonads [2]. Geographic variation has been found in this species even within Scotland, with the size at the onset of maturity varying between distinct populations from locations with different ecological features [11]. Seasonal variation in the *Nephrops* tissue composition could also be expected, as sexually mature females develop enlarged gonads (ovaries) during the spring that could potentially contribute large amounts of lipid and yolk products to any head waste harvested over the summer months [5]. These effects may be further exacerbated by the greater prevalence of females in summer-time trawl catches, due to a seasonal change in their emergence behaviour that makes them more susceptible for capture [12,13,14]. 

This study aimed to determine the amount of oil that can be extracted from the head waste produced by the *Nephrops* fishery, taking into consideration both Geographical and seasonal variations. In addition, this study compared the lipid composition (lipid class analysis and fatty acid composition) of *Nephrops* head waste with the lipid that can be extracted from commercially available krill *E. superba*. 

## 2. Results

### 2.1. Geographical Variation in Catch Composition and Lipid Content in Nephrops Head Waste

In order to evaluate the impact of geographical location on lipid content, head waste samples from *Nephrops* collected in three fishing different grounds were analysed. As shown in Table 1, clear differences in the *Nephrops* catch composition and lipid content in *Nephrops* head waste were found according to geographical location of the samples (Clyde Sea area vs. Minches vs. coast in southern Iceland) (Table 1). The percentage of females in the catch was similar in the Minch (66%) and the Clyde (70%) but was considerably lower in Iceland (42%). *Nephrops* head waste from Iceland contained significantly lower values of total lipid (Table 1). This trend was also supported by lower detected lipid levels in the hepatopancreas and gonads of the females in Iceland samples. Furthermore, the percentage of females and females showing mature gonads (stage 3/4) from Iceland was the lowest among the sites studied. 

### 2.2. Seasonal Variation in Nephrops Head Waste in the Clyde Sea Area

As the Clyde Sea area exhibited the highest values of lipid, further analysis was undertaken to assess seasonal variations in this particular fishing ground.

#### 2.2.1. Seasonal Variability in *Nephrops* Catch Composition and Lipid Content

**Figure 1:**
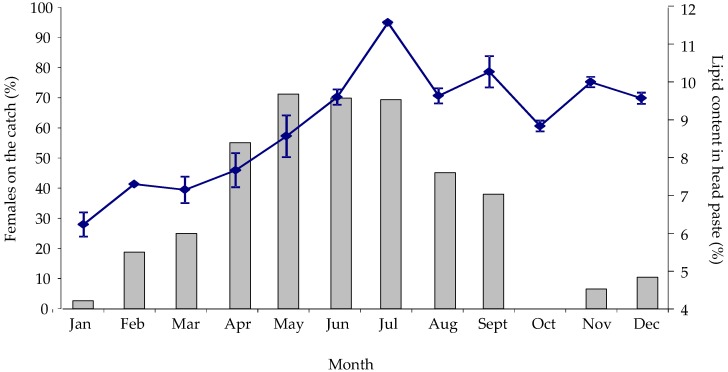
Percentage of female *Nephrops* in a random sample taken from the trawls (bars) and lipid content (% of dry weight) in the head pastes of trawled *Nephrops* (mixed sexes) (blue line) in the Clyde Sea area monthly for over a period of a year. Values are the mean ± standard error (*n* = 3).

The sex composition of *Nephrops* caught by otter trawl in the Clyde Sea area showed a strong seasonal pattern. The proportion of female *Nephrops* caught peaked in May (Figure 1). Similarly, the percentage of lipid present in the *Nephrops* head waste had a strong seasonal component. In this case, the maximum percentage of lipid was reached in July, two months later than the observed peak in the female sex ratio (Figure 1). Nevertheless, there was a significant correlation between lipid percentage in the head waste and female composition of the catch (*F*_1,35_ = 10.13, *p* = 0.003). Lipid percentage varied significantly with season (*F*_11,35_ = 13.55, *p* < 0.001), indicating that, in the Clyde Sea area the head wastes contained a significantly higher percentage of lipid in the summer months than in the winter months. The total body weight of caught females was significantly higher in summer (*p* < 0.05), while in contrast total body weight of males caught in the trawls did not vary significantly throughout the year (Table 2).

#### 2.2.2. Body Indices and Lipid Content in *Nephrops* Tissue Samples

To further investigate the processes influencing the amount of lipid in *Nephrops* head waste, hepatosomatic index (HSI), gonadosomatic index (GSI), the lipid content in the hepatopancreas, female gonad and ovary maturation stage (see stages in Section 4.5) were recorded (Table 2). HSI was highest for males in autumn and for females in spring. Lipid content in the hepatopancreas samples varied significantly throughout the year (*F*_1,36_ = 28.763, *p* < 0.001) (Table 2). In males, lipid contents were lower in spring compared to the other seasons, while in females lower lipid contents were obtained in spring and also summer compared to colder seasons. The hepatopancreas lipid content varied significantly between males and females (*F*_1,36_ = 8.637, *p* = 0.006), likely due to the overall higher lipid content in female hepatopancreas samples in all seasons, except in summer (Table 2).

#### 2.2.3. Fatty Acid Composition in *Nephrops* Head Waste

The fatty acid composition of head waste at different times of the year from the Clyde Sea area is presented in Table 3. Total monounsaturated fatty acids (MUFAs) accounted for approximately 40% of *Nephrops* head waste oils, while total PUFAs accounted for approximately 35% of the total fatty acids. The *n*-3 PUFAs content in the oil varied significantly depending on the time of the year (*F*_3,4_ = 13.144, *p* = 0.015) with the highest concentrations of *n*-3 PUFAs, in particular 20:5*n*-3 (EPA, 15.5%) and 22:6*n*-3 (DHA, 8.4%), recorded in summer. DHA did not exhibit significant seasonal variation (*F*_3,4_ = 1.8997, *p* = 0.271), whereas EPA was significantly higher in the summer (10.5% and 15.5% in winter and summer, respectively) (*F*_3,4_ = 7.5943, *p* = 0.040).

### 2.3. Comparison of Lipid Class and Fatty Acid Composition between Nephrops Head Waste and Krill Oil

The nutritional quality of *Nephrops* head waste oil was then compared to the commercially valuable oil from the krill species *E. superba*. Lipid class and fatty acid composition according to lipid classes of *Nephrops* head waste samples and krill oil samples were analysed. *Nephrops* head waste samples used for this analysis were collected from the Clyde Sea area during the summer when lipid content is at its highest. Similar to the values obtained in samples from the seasonal trial, *Nephrops* head waste collected by commercial processing contained 10.7% total lipid. Regarding lipid class composition of *Nephrops* oil, similar percentages of total neutral (68.2%) and polar (31.8%) lipids were obtained when compared with krill oil (Table 4). Within the neutral lipids, TAG was the most abundant fraction in both oils (Table 4). Within the polar lipids, phosphatidylcholine (PC) was found to be the most abundant polar lipid class in both *Nephrops* head waste and krill oils (Table 4). Strong differences were found between *Nephrops* head waste oil and krill oil when comparing the fatty acid profiles of the lipid class fractions (Table 5). In the neutral lipids, krill oil contained 45.1% and 42.5% of total fatty acids as saturated and monounsaturated fatty acids, respectively (Table 5), with relatively low levels of both *n*-6 and *n*-3 PUFAs (11.1%). While the neutral lipids from *Nephrops* oil contained similar levels of monounsaturated fatty acids (45.5%) to krill oil, it contained lower levels of saturated fatty acids (21.3%) and higher levels of PUFAs (32.5%). In particular, both EPA (15.0% of total neutral lipids) and DHA (8.3%) were the most abundant PUFA within the neutral lipids from *Nephrops* head oil (Table 5). Within the polar lipids, both *Nephrops* and krill oils contained similar levels of saturated fatty acids (21.3% and 26.5%, respectively), although *Nephrops* oil had relatively higher levels of monounsaturated fatty acids (30.0%) compared to krill oil (19.3%). Polar lipids from both oils contained PUFA and *n*-3 PUFA in particular, as the most abundant type of fatty acids. Both EPA and DHA showed the highest contents in both oils’ polar lipids, with slightly higher levels found in the krill oil (26.1% and 20.8%, respectively) compared to the *Nephrops* oil (22.0% and 15.4%, respectively) (Table 5).

## 3. Discussion

Given recent amendments to the Common Fisheries Policy made by the European Union [3], innovative approaches to the utilisation of fisheries discards and waste are urgently needed. In the UK, approximately 25,000 tonnes of *Nephrops* are landed each year, with 45% of this catch weight (11,000 tonnes) destined to the scampi market. This results in nearly 6500 tonnes of wasted heads per year in the UK fishery alone, as the head portion accounts for 2/3 of body weight [15]. In the present study, we analysed the amount and composition of the lipid that could be extracted from wasted *Nephrops* heads, which are currently disposed of after tailing procedures at sea. As shown in this study, this wasted *Nephrops* head material contains significant amounts of lipid (ranging from 6% to 11.5% lipid on a dry weight basis). These values are lower than those reported in some other crustacean species such as krill (12%–50% lipid dry weight) [6] but higher than the lipid content reported in other shell-rich wastes such as Northern shrimp shells (<1% lipid dry weight) [16]. The economic viability of extracting such oil should therefore now be evaluated. From a natural resource perspective, previous studies have indicated that the lipid content of krill can vary considerably depending on species, age and time between capture and freezing [17]. We therefore aimed to uncover the particular variables that account for variation in the lipid content of *Nephrops.*

Geographical differences play a prominent role in the amount of lipid that could be extracted from *Nephrops* head waste. In the June *Nephrops* sampling, females comprised 70% of the catch in the Clyde Sea area but only 40% of the catch in Iceland. Ovaries from those females sampled in Iceland were shown to be at an earlier maturation stage, possibly indicating a delay in female emergence, food availability and gonad maturation. In addition, cold-water *Nephrops* around Iceland have been found to breed on a biennial cycle, unlike the annual cycle in more southern locations like Scotland [18,19]. From these data, it is not possible to eliminate any of these factors as only one sampling could be performed. However, through the data from the Clyde Sea area, it is clear that the female body concentration of lipid will peak in summer months, likely due to the fact that they maximise storage of lipids in their hepatopancreas and maintain high percentages of lipid in a larger gonad at this time of the year.

The amount of lipid in *Nephrops* head paste also showed a strong seasonal pattern, with percentages ranging from 6.2% ± 0.3% in January to 11.5% ± 0.4% in July (dry basis). This seasonality in the amount of lipid extracted has also been observed in other crustacean sources such as North Atlantic krill [20]. This pattern can be explained, as shown in the present study, by several factors. Firstly, the seasonality of spawning and moulting in *Nephrops* females has previously been described for different regions in Scotland [2,21,22]. In the Clyde Sea area, females tend to emerge from their burrows to moult and mate in early summer and are therefore more prevalent in the catches during the summer months [14,23]. This pattern was also observed in the sampling performed in the present study: females became more predominant (>50%) in the trawl catches from April to July and decreased afterwards to become very low during the winter months (November to March). However, the amount of lipids in *Nephrops* head paste cannot be explained solely by the emergence of females since lipid percentage in the head paste peaked in July, which is approximately two/three months after the emergence of females, which started in spring (April). Changes in the weight and lipid content of the soft tissues present in the cephalothorax may also have been involved. GSI increased from 2.9% in spring to 6.4% in the summer with a concomitant increase in the lipid content in the hepatopancreas, although not for HSI, for both males and females. Similar trends in the lipid percentage, HSI and GSI and in the hepatopancreas have been reported in other studies [5]. While in general GSI has been shown to clearly change seasonally in relation to the maturation state of the female gonads [5,24], HSI and the percentage of lipid in the hepatopancreas has been shown to be more variable. For instance, in some studies [25] lipid content in the hepatopancreas has been reported not to change significantly with season, while in other studies conducted in the Clyde Sea area [26] the lowest levels were obtained in the spring, which is similar to the present study. The earlier increase in HSI observed in females should then be explained by an increase in other constituents, such as water content [23] rather than an increase in lipid content. Interestingly, we also observed seasonality in the content of (*n*-3) PUFA in *Nephrops* paste, with samples collected in summer having higher contents of EPA (DHA remained fairly constant) compared to those from winter. Considering the herein observed prevalence of sexually mature females during summer, it is reasonable to suggest that the observed increase of EPA during summer is likely due to an accumulation of EPA in female gonads for reproductive purposes, consistent with this essential fatty acid being regarded as playing a pivotal role in crustacean reproductive physiology [27].

Lipid extracted from *Nephrops* exhibited high levels of (*n*-3) PUFAs, which represent some of the most valuable components in krill oil. Krill oil and its associated products have become the basis of a valuable industry [6,28]. Studies indicate that (*n*-3) PUFAs account for approximately 20%–25% of the total fatty acids in krill oil, which is comparable to values obtained in this study for both krill and *Nephrops* head waste oil (approximately 23%) [29]. The primary advantage of krill oil over fish oil is its enhanced absorption, which has been attributed to the fact that in krill oil the *n*-3 PUFAs are mainly incorporated into phospholipids (PL) rather than triacylglycerols (TAG) [30]. In the present study, we observed that the incorporation of fatty acids such as DHA and EPA into the polar lipid fraction is a unique feature of krill oil, as in *Nephrops* oil *n*-3 PUFAs were also substantially represented in the neutral fraction.

In conclusion, using various analytical methods, the amount and composition of the oil that can be obtained from *Nephrops* head waste has been determined. The amount and composition of the extracted lipid varies considerably with the geographic location of fishing grounds and with season. The biological basis for these variations is a combination of seasonal changes in the sex ratios of the catches, the size of the female gonad and the composition of the hepatopancreas. These results highlight the importance in understanding sources of variability in order to develop a cost-effective harvesting strategy that maximises yields of useful compounds from any natural resource. Results indicate that the extraction of lipid from *Nephrops* wasted heads, especially during the summer months, could provide an alternative source of income for the fishery, increase its sustainability and meet suggested future requirements on discards within the Common Fisheries Policy reforms in the European Union. Further work to assess the economic feasibility of such a process, in combination with the extraction of other compounds in an industrial setting, would now be needed.

## 4. Materials and Methods

### 4.1. Geographic Variation in Catch Composition and Lipid Content in Nephrops Head Waste

During the month of June, *Nephrops* from three common fishing grounds were collected by otter trawl using 70 mm nets. These locations were (1) a transect located in the Clyde Sea area, in the Largs-Fairlie Channel (55′51.351 N/4′54.424 W to 55′48.979 N/4′54.055 W) (2) a transect from the Minch, off Stornoway in Northern Scotland (58′02.716 N/6′15.249 W to 57′57.195 N/6′15.742 W) and (3) a transect from the area of Háfadypí to the west of Heimaey, Iceland (63′15.817 N/20′01.242 W to 63′18.312 N/19′57.359 W).

### 4.2. Seasonality Variation in Nephrops Head Waste in the Clyde Sea Area

*Nephrops* specimens used to determine seasonal variations were caught by otter trawl using 70 mm nets on a fishing ground off the west coast of Scotland, in the Clyde Sea area transect detailed above. Sampling was conducted monthly for a one-year period by the research vessel RV Aplysia from the UMBSM research station. 

### 4.3. Initial Sample Processing and Transport of Samples

During all *Nephrops* collections, once the trawl nets were emptied onto the deck, *Nephrops* were separated from the catch and a random grab sample of 70 *Nephrops* with a carapace length (CL) <40 mm was taken for use in the studies of head waste lipid composition. Lobsters with a CL >40 mm would be commonly sold as whole animals, rather than being “tailed”, so were not used in this study [15]. Furthermore, 20 males and 20 females were also selected for use in analyses of individual body organs (hepatopancreas and female ovary studies). Following sorting, all samples were washed with running seawater, declawed, and placed on ice (Clyde Sea area) or frozen (samples from the Minch and Iceland) for transport back to the University of Glasgow.

### 4.4. Comparison of Nephrops Head Waste Oil versus Oil Extracted from Krill

In order to characterise the nutritional composition and potential use of *Nephrops* head waste as a source material for high value oil, samples of *Nephrops* head waste were compared to the oil extracted from krill, which has a recognised value. *Nephrops* head waste was collected from a *Nephrops* processing plant, Angelbond Ltd. (Glasgow, UK), which sources its specimens from vessels operating in the Clyde Sea area. Three independent samples were collected directly from the processing plant over the months of June and July. From each sample *Nephrops* head paste was generated from approximately 70 animals (mean CL 31.4 mm) as explained in Section 4.5. For comparison, krill paste was obtained from Tharos (Santiago, Chile) and processed in the same way.

### 4.5. Head Waste and Tissue Sample Preparation

From each random grab sample of *Nephrops*, approximately 70 animals were used to generate a head homogenate or “paste” sample, equivalent to other commercial head waste products [15]. The sex of each animal in this random sub-sample was recorded and used to calculate the female sex ratio of the trawls in each month. Head waste containing the entire cephalothorax, including walking legs, eyes and all internal organs (including the hepatopancreas and gonads) was blended to create a fine paste using a knife-mill (Grindomix GM 200, Retsch, UK). The resulting head paste was placed in a container and frozen at −25 °C.

Hepatopancreas and female gonad tissues were dissected from the 20 male and 20 female *Nephrops*. Each individual was weighed (without claws) to determine the body weight. For both male and female *Nephrops* samples, the hepatopancreas tissue was collected and weighed. The hepatopancreas tissue was pooled together by sex and frozen at −25 °C. For each female sample, the ovaries were collected and weighted. The stage of maturation was then recorded following the scale used by Farmer [31], which is based on the ovary colour (stage 0: white ovary, not yet sexually mature or have gone under resorption postmoult; stage 1: cream ovary, initial oocyte development; stage 2: pale green ovary, intermediate oocyte development; stage 3/4: dark green ovary, maximum oocyte development and stage 5: mottled green/cream ovary almost spent). The ovaries from all of the female samples collected were then pooled and frozen at −25 °C. From these data, the hepatosomatic index (HSI) and the gonadosomatic index (GSI) were calculated using the following formulas:

$$\mathrm{HSI}=\frac{\mathrm{Hepatopancreas}\;\mathrm{wet}\;\mathrm{weight}\;\left(g\right)}{\mathrm{Body}\;\mathrm{wet}\;\mathrm{weight}\;\left(g\right)}\;\times 100$$

$$\mathrm{GSI}=\frac{\mathrm{Ovaries}\;\mathrm{wet}\;\mathrm{weight}\;\left(g\right)}{\mathrm{Body}\;\mathrm{wet}\;\mathrm{weight}\;\left(g\right)}\;\times 100$$

### 4.6. Total Lipid Extraction and Lipid Class Analysis

All chemicals except otherwise stated were from Fisher Scientific, Loughborough, UK. Total lipids from *Nephrops* head waste paste, krill paste and tissue samples collected from trials detailed in Section 4.1, Section 4.2, Section 4.3 and Section 4.4 were extracted according to the Folch extraction method [32]. Prior to extraction, head paste, hepatopancreas, and female gonad samples were freeze-dried. Extracted lipids were then resuspended in chloroform: methanol (2:1, *v*/*v*) with butylated hydroxytoluene (BHT) (Sigma-Aldrich, Dorset, UK) (0.01%, *w*/*v*) to a final concentration of 10 mg·mL^−1^ and frozen at −25 °C until further use. Three replicate lipid extractions were performed on each pooled *Nephrops* head and krill pastes and tissue samples.

Lipid classes were separated for either preparative or analytical purposes. For preparative purposes, total lipids were loaded onto 20 cm × 20 cm thin-layer chromatography plates (Merck, Darmstadt, Germany) and eluted with a solvent mixture composed of isohexane:diethyl ether:glacial acetic acid (80:20:1, *v*/*v*/*v*). After visualizing with 2.7 dichlorofluorescein total neutral and total polar lipids were recovered by eluting the scrapped off silica in chloroform: methanol (2:1, *v*/*v*) until further use for fatty acid analysis (Section 4.7). Lipid class analyses were determined in total lipid samples using high-performance thin-layer chromatography (HPTLC) as described in [33]. Lipid classes were visualised by charring at 160 °C for 15 min after spraying with 3% (*w*/*v*) aqueous cupric acetate containing 8% of phosphoric acid and quantified by densitometry using a CAMAG-3 TLC Scanner (Version Firmware 1.14.16; CAMAG, Muttenz, Switzerland) with winCATS Planar Chromatography Manager. Identification of individual classes was confirmed by comparing Rf values of known standards run alongside samples.

### 4.7. Fatty Acid Analysis: FAME Preparation and Gas Chromatography 

Fatty acid methyl esters (FAMEs) from total lipid or total neutral and polar lipid fractions were prepared by acid-catalised transesterification of total lipid according to the method of Christie [34]. FAMEs were separated and quantified by a GLC (Fisons GC-8160, Thermo Scientific, Milan, Italy) equipped with a 30 mm × 0.32 mm i.d. × 0.25 μm ZB-wax column (Phenomenex, Cheshire, UK), fitted with “on column” injection and flame ionisation detection. Hydrogen was used as the carrier gas and temperature programming was from 50 to 150 °C increasing at 40 °C·min^−1^, followed by a gradient of 2 °C·min^−1^ to a final temperature of 230 °C. Individual FAMEs were identified by comparison with known standards (Supelco 37 FAME mix, Sigma-Aldrich Ltd., Poole, UK) and published data [33]. Data were collected and processed using Chromcard for Windows Version 1.19 (Thermoquest Italia SpA, Milan, Italy).

### 4.8. Statistical Analysis

Results are presented as mean and standard error of the mean (SEM) (body indices *n* = 20; lipid analysis *n* = 3). Seasonal variation in the HSI, GSI, percentage of lipid, and total lipid content of the tissues were assessed using generalised linear models (GLMs) in the statistical software R version 2.15.0 [35]. GLMs were also performed to determine if differences between male and female HSI, percentage lipid of the hepatopancreas, and total lipid content of the hepatopancreas. The normality, linearity and homogeneity of residuals were confirmed by inspection of residual-fit and quantile-quantile plots. Homogeneity of variances was assessed by Levene’s test. Data from seasonal and Geographical variation trials were also compared by one-way analysis of variance (ANOVA), with post hoc comparisons using Tukey’s test. *p* < 0.05 were considered significant.

## Figures and Tables

**Table 1:** Catch composition, body indices, gonad maturation stage in females and lipid content in pooled samples from *Nephrops* head waste material and relevant tissues (male and female hepatopancreas) and female gonads in samples collected from fishing grounds located in different Geographical locations. Values represent mean ± SEM. Number of replicates in body indices is *n* = 20 and lipid composition is *n* = 3. Different letters indicate values that are significantly different according to Geographical location (*p* < 0.05).

	Winter	Spring	Summer	Autumn
**Carapace Length (mm)**				
Male	33.0 ± 0.8	32.6 ± 1.4	32.6 ± 1.3	32.3 ± 0.8
Female	27.2 ± 0.5 ^a^	31.6 ± 1.1 ^a^	35.5 ± 1.9 ^b^	30.8 ± 1.8 ^a^
**Body weight (g)**				
Male	18.7 ± 1.6	20.4 ± 2.6	18.7 ± 2.1	17.1 ± 1.3
Female	10.6 ± 0.6 ^a^	18.7 ± 2.3 ^b^	25.8 ± 4.4 ^c^	19.6 ± 3.7 ^b^
**HSI (%)**				
Male	5.1 ± 0.2 ^a^	5.5 ± 0.2 ^a^	5.0 ± 0.3 ^a^	7.1 ± 0.7 ^b^
Female	5.2 ± 0.3 ^a^	6.1 ± 0.4 ^b^	5.5 ± 0.4 ^ab^	5.1 ± 0.4 ^ab^
**GSI (%)**				
Female	0.7 ± 0.1 ^a^	2.9 ± 0.3 ^b^	6.4 ± 0.5 ^c^	2.5 ± 1.0 ^ab^
**Stage 3/4 gonads (%)**				
Female	0	45.4	86.4	23.1
**Lipid content (%)**				
Hepato Male	46.4 ± 0.2 ^a^	28.5 ± 1.2 ^b^	46.2 ± 0.6 ^a^	47.6 ± 0.5 ^a^
Hepato Female	56.1 ± 0.7 ^a^	41.9 ± 1.3 ^b^	46.4 ± 1.6 ^b^	58.4 ± 1.6 ^a^
Gonad (Female)	17.4 ± 1.1 ^a^	23.4 ± 0.5 ^b^	26.4 ± 0.8 ^b^	24.3 ± 0.50 ^b^

**Table 2:** Carapace length, body weight, body indices, gonad maturation stage in females and lipid content in pooled samples from relevant tissues in *Nephrops* collected in the Clyde Sea area at different times of the year: Winter-December; Spring-April; Summer-July and Autumn-September. Values represent mean ± SEM. Number of replicates in body indices is *n* = 20 and lipid composition is *n* = 3. Different letters indicate values that are significantly different according to season (*p* < 0.05).

	Sampling Location		
	Clyde	Minch	Iceland
**Females in the catch (%)**	70	66	42
**Total lipid head paste (%)**	9.6 ± 0.2 ^c^	7.7 ± 0.2 ^b^	5.3 ± 0.1 ^a^
**HSI (%)**			
Males	5.5 ± 0.3 ^b^	3.3 ± 0.9 ^a^	3.2 ± 0.2 ^a^
Females	5.8 ± 0.3 ^c^	3.7 ± 0.2 ^b^	2.11 ± 0.2 ^a^
**GSI (%)**			
Females	4.1 ± 0.4 ^b^	3.9 ± 1.4 ^b^	2.6 ± 0.3 ^a^
**Females at gonad stage 2**	17	14	58
**Females at gonad stage 3/4**	74	81	0
**Lipid content (%)**			
Hepato Males	37.7 ± 0.4 ^a^	46.3 ± 0.1 ^b^	43.3 ± 1.6 ^b^
Hepato Females	45.0 ± 0.5 ^c^	38.4 ± 1.7 ^b^	31.3 ± 0.1 ^a^
Gonad (Females)	25.9 ± 0.6 ^b^	23.9 ± 0.5 ^b^	13.7 ± 0.5 ^a^

**Table 3:** Fatty acid composition (% of total fatty acids) in oil extracted from *Nephrops* head waste sampled from the Clyde Sea area at different times of the year; n.d. not detected.

Fatty Acid (%)	Winter	Spring	Summer	Autumn
14:0	1.1	1.4	1.6	1.7
15:0	0.7	0.6	0.6	0.6
16:0	12.5	12.1	12.6	13.6
18:0	4.2	3.8	3.9	4.1
19:0	0.2	0.2	0.2	0.2
20:0	0.4	0.4	0.2	0.4
22:0	0.1	0.4	n.d.	0.4
**Total saturated**	**22.9**	**22.2**	**22.0**	**24.4**
16:1*n*-9	0.2	0.2	0.3	0.3
16:1*n*-7	5.6	6.6	7.3	6.2
17:1	0.8	0.6	0.6	0.6
18:1*n*-9	14.8	13.3	13.0	13.0
18:1*n*-7	8.3	7.8	7.8	8.3
19:1	0.3	0.4	0.4	0.4
20:1*n*-11	4.1	3.1	3.4	3.1
20:1*n*-9	3.8	3.1	2.6	3.1
20:1*n*-7	5.7	4.5	4.5	4.2
22:1*n*-11	1.0	1.2	0.5	0.9
22:1*n*-9	0.6	0.7	0.5	0.9
22:1*n*-7	1.3	1.0	0.8	1.0
**Total monounsaturated**	**46.5**	**42.4**	**41.8**	**42.1**
18:2*n*-6	0.7	0.7	0.8	0.9
18:3*n*-6	0.2	0.2	0.1	n.d.
20:2*n*-6	1.2	1.1	1.0	1.0
20:3*n*-6	0.2	0.1	0.1	0.2
20:4*n*-6	4.0	3.8	3.8	3.4
22:4*n*-6	0.7	0.6	0.8	0.8
22:5*n*-6	0.6	0.5	0.4	0.5
**Total *n*-6 PUFA**	**7.6**	**7.0**	**7.1**	**6.7**
18:3*n*-3	0.2	0.2	0.2	0.3
18:4*n*-3	0.3	0.7	0.8	0.7
20:3*n*-3	0.2	0.2	0.2	0.2
20:4*n*-3	0.3	0.4	0.4	0.4
20:5*n*-3	10.5	13.9	15.5	13.1
22:5*n*-3	1.8	1.7	2.1	2.0
22:6*n*-3	8.5	9.6	8.4	8.5
**Total *n*-3 PUFA**	**21.7**	**26.5**	**27.6**	**25.2**

**Table 4:** Lipid class (% of total lipid) in oil extracted from *Nephrops* head waste from the Clyde Sea area (summer sample) compared to oil extracted from commercially available krill. Values represent mean ± SEM (*n* = 3 *Nephrops* and *n* = 2 krill); n.d. not detected.

Lipid Class (% Total Lipid)	*Nephrops*	Krill
Sterol esters	3.85 ± 0.01	5.60 ± 0.55
Triacylglycerols	33.93 ± 2.52	37.99 ± 1.77
Free fatty acids	14.99 ± 0.43	9.90 ± 0.44
Cholesterol/sterols	14.89 ± 0.89	10.53 ± 0.58
Unknown neutral lipid ¶	0.57 ± 0.17	3.48 ± 0.11
**Total neutral lipids**	**68.23 ± 1.01**	**67.49 ± 0.30**
Monogalactosyldiacylglycerols	n.d.	n.d.
Unknown glycolipid	n.d.	n.d.
Digalactosyldiacylglycerols	n.d.	n.d.
Unknown polar lipid *	n.d.	1.22 ± 0.05
Phosphatidylethanolamine	7.42 ± 0.05	6.70 ± 0.11
Phosphatidic acid/Phosphatidylglycerol/cardiolipin	1.50 ± 0.06	1.52 ± 0.18
Phosphatidylinositol	2.42 ± 0.03	1.29 ± 0.11
Phosphatidylserine	2.65 ± 0.20	0.90 ± 0.17
Phosphatidylcholine	14.04 ± 0.76	18.01 ± 0.72
Sphingomyelin	1.15 ± 0.18	n.d.
Lysophosphatidylcholine	1.11 ± 0.15	2.49 ± 0.26
Pigmented material	1.48 ± 0.06	0.39 ± 0.13
**Total polar lipids**	**31.77 ± 1.01**	**32.51 ± 0.30**

* Possibly Sulfolipid; ¶ Possibly diacylglycerol.

**Table 5:** Fatty acid composition according to lipid classes (% of total lipid class) in oil extracted from *Nephrops* head waste from the Clyde Sea area (summer sample) compared to oil extracted from commercially available krill; n.d. not detected.

Fatty Acid (%)	Neutral Lipids		Polar Lipids	
	*Nephrops*	Krill	*Nephrops*	Krill
14:0	3.3	18.7	1.0	2.2
15:0	0.7	0.6	0.6	0.4
16:0	12.7	23.7	12.6	21.8
18:0	3.4	1.8	6.2	1.6
19:0	0.5	n.d.	0.2	0.2
20:0	0.3	0.2	0.3	n.d.
22:0	0.2	n.d.	n.d.	0.2
**Total saturated**	**21.3**	**45.1**	**21.3**	**26.5**
16:1*n*-9	0.4	1.0	0.8	0.2
16:1*n*-7	9.6	12.5	5.3	1.9
17:1	0.5	0.4	0.5	0.2
18:1*n*-9	13.1	18.3	11.7	7.9
18:1*n*-7	7.2	8.1	5.7	6.8
19:1	0.4	n.d.	0.3	n.d.
20:1*n*-11	3.3	n.d.	1.3	n.d.
20:1*n*-9	3.2	1.4	2.1	0.6
20:1*n*-7	4.6	0.5	2.0	0.2
22:1*n*-11	2.0	n.d.	0.4	n.d.
22:1*n*-9	0.9	0.4	n.d.	1.0
24:1*n*-9	0.3	n.d.	n.d.	0.4
**Total monounsaturated**	**45.5**	**42.5**	**30.0**	**19.3**
18:2*n*-6	1.1	1.3	1.2	2.3
18:3*n*-6	0.2	0.2	0.3	n.d.
20:2*n*-6	1.0	n.d.	0.8	n.d.
20:3*n*-6	0.2	n.d.	n.d.	n.d.
20:4*n*-6	2.5	0.1	4.0	1.0
22:4*n*-6	0.5	n.d.	0.5	0.2
22:5*n*-6	0.4	n.d.	0.3	0.7
**Total *n*-6 PUFA**	**5.9**	**1.7**	**7.1**	**4.1**
18:3*n*-3	0.3	0.5	0.4	1.0
18:4*n*-3	0.9	2.1	0.8	1.5
20:3*n*-3	0.2	n.d.	n.d.	n.d.
20:4*n*-3	0.4	0.1	0.4	0.4
20:5*n*-3	15.0	4.3	22.0	26.1
22:5*n*-3	1.4	0.1	2.1	n.d.
22:6*n*-3	8.3	2.3	15.4	20.8
**Total *n*-3 PUFA**	**26.6**	**9.4**	**41.3**	**49.8**
